# Hepatoprotective activity of ethanolic extract of *Salix subserrata* against CCl_4_-induced chronic hepatotoxicity in rats

**DOI:** 10.1186/s12906-016-1238-2

**Published:** 2016-07-29

**Authors:** Ahmed Wahid, Ashraf N. Hamed, Heba M. Eltahir, Mekky M. Abouzied

**Affiliations:** 1 Department of Pharmacology and Toxicology, College of Pharmacy, Taibah University, Medina, Kingdom of Saudi Arabia; 2 Biochemistry Department, Faculty of Pharmacy, Minia University, 61519 Minia, Egypt; 3 Pharmacognosy Department, Faculty of Pharmacy, Minia University, 61519 Minia, Egypt

**Keywords:** *Salix Subserrata*, Carbon tetrachloride, Hepatoprotective, Silymarin, Liver injury, NF**k**B, TNF-α

## Abstract

**Background:**

The liver performs diverse functions that are essential for life. In the absence of reliable liver protective drugs, a large number of natural medicinal preparations are used for the treatment of liver diseases. Therefore the present study aims to investigate the hepatoprotective effects of *Salix subserrata* Willd flower ethanolic extract (SFEE) against carbon tetrachloride (CCl_4_)-induced liver damage.

**Methods:**

Rats were divided into 4 groups of 10 animals each. Group I served as the normal healthy control, groups II rats were intoxicated with CCl_4_ i.p. (0.8 ml/kg body weight CCl_4_/olive oil, twice weekly for 9 weeks), group III rats received CCl_4_ i.p. and SFEE orally (150 mg/kg daily) and group IV rats received CCl_4_ i.p. and Silymarin orally (100 mg/kg, daily). The hepatoprotective potential of SFEE in rats was evaluated by measuring the protein levels of two inflammatory biomarkers; tumor necrosis factor-alpha (TNF-α) and nuclear factor kappa-B (NF-**k**B) in addition to other liver biomarkers. Histopathological changes in the liver were assessed using hematoxylin and eosin staining (HE).

**Results:**

The administration of SFEE showed hepatic protection at an oral dose of 150 mg/kg. SFEE significantly reduced the elevated serum levels of intracellular liver enzymes as well as liver biomarkers in comparison to CCl_4−_ intoxicated group. Notably, SFEE significantly reduced the expression levels of TNF-α and NFkB proteins compared to their levels in CCl_4_ intoxicated group. These findings were confirmed with the histopathological observations, where SFEE was capable of reversing the toxic effects of CCl_4_ on liver cells compared to that observed in CCl_4_-intoxicated animals.

**Conclusion:**

Our results show that SFEE has potential hepatoprotective effects at 150 mg/kg. These effects can be regarded to the antioxidant and anti-inflammatory properties of the extract.

## Background

The liver performs diverse functions that are essential for life. It directly receives, processes and stores materials absorbed from the digestive tract. It has been shown before that oxidative stress and inflammation are leading causes of liver diseases. Carbon tetrachloride (CCl_4_) administration can induce chronic liver injury in rats. It is therefore considered as the experimental model of choice for liver injury [1, 2]. The liver damaging effect of CCl_4_ is explained by its ability to produce trichloromethyl free radicals and reactive oxygen species (ROS) after being metabolized by cytochrome P450. These metabolites initiate a lipid peroxidation chain reaction and eventually lead to many chronic diseases including liver injury [3, 4]. Therefore, this model has been widely used for evaluating the therapeutic effect of many hepatoprotective drugs [5].

It has been reported before that antioxidants prevent oxidative damage caused by free radicals and can thereby reduce the risk of liver diseases [6]. Herbal medicines have been used extensively for decades for the treatment of many diseases. Indeed, natural products continue to be important sources for the development of many drugs to treat a wide variety of diseases such as cancer and liver disease among others [7].

*Salix caprea* is a plant that belongs to the Salicaceae family. The flowers of *Salix caprea* have been reported to possesses anti-inflammatory properties, which were demonstrated using the human red blood cell (HRBC) membrane stabilization method [8]. Other parts of the Salix species, such as the bark, were shown to possess the same anti-inflammatory properties as the flowers [9, 10]. The free radical scavenging ability of Salix extract was related to the large number of polyphenolic compounds that were detected in such extract. It was shown that *Salix caprea* extract scavenged 2,2-diphenyl-1-picrylhydrazyl (DPPH), superoxide dismutase (SOD) and hydrogen peroxide (H_2_O_2_) as a result of its antioxidant properties [11]. *Salix subserrata* Willd is another species of the same family. It is a shrub or a tree of 2 to 10 m high that is usually found in moist locations, often beside streams, rivers, lakes and other surface waters throughout Africa (Egypt, Sudan, Libya, Gambia, and Zambia) [12, 13].

*Salix* is used in folk medicine since ages for the treatment of different ailments in human and animals as well. It is used to relief fever, headaches, constipation and stomachache. Also the leaves were reported to be effective in treatment of rabies when used with milk [14]. Phytochemical analysis of the plant revealed the presence of several active ingredients that include flavonoids and phenolic compounds in addition to other compounds. Flavonoids include rutin, luteolin-7-glucoside, quercetrin, and quercetin whereas phenolic compounds include catechins and salignin [15, 16].

In the present study, we aim to investigate the hepatoprotective effects of ethanolic extract of *S. subserrata* flower against CCl_4_-induced oxidative stress and its role in the alleviation of lipid peroxidation and restoration of TNF-α and NF-**k**B levels and liver enzymes activities.

## Methods

### Plant materials and preparation of SFEE

The flowers of *S. subserrata* were collected in March 2013 from the campus of Minia University, Minia, Egypt. It was identified by Dr. Magdy H. A. Ahmed, Plant and Agricultural Microbiology Department, Faculty of Science, Minia University, Minia, Egypt. A voucher specimen of the plant under the number Mn-Ph-Cog-009 was deposited in the Herbarium of Pharmacognosy Department, Faculty of Pharmacy, Minia University, Minia, Egypt. The flowers were air-dried and reduced to fine powder suitable for extraction. One kilogram of the air-dried fine powder was macerated in ethanol until exhaustion (4 L four times with intervals of 7 days) and then concentrated under reduced pressure until dryness using a rotary evaporator to yield 30 g the ethanolic extract.

### In vitro antioxidant activity of Salix subserrata

The antioxidant activity of *Salix subserrata* ethanolic extract was estimated using DPPH as previously outlined [17]. Briefly, different concentrations of SFEE (50, 100, and 150 ug/mL) were mixed with DPPH solution (4 mg/50 mL methanol) and the decrease in the absorbance of DPPH was measured after 30 min spectrophotometrically at 517 nm. The absorbance of DPPH in MeOH alone served as blank. Similar concentrations of ascorbic acid (50, 100, and 150 ug/mL) were used as standard. Determinations were performed in triplicate. The following formula was used to calculate the percentage of inhibition:

$$ \%\kern0.5em \mathrm{of}\kern0.5em \mathrm{inhibition}=100\times \left(1-\left(\mathrm{Absorbance}\kern0.5em \mathrm{with}\kern0.5em \mathrm{compound}/\mathrm{Absorbance}\kern0.5em \mathrm{of}\kern0.5em \mathrm{the}\kern0.5em \mathrm{blank}\right)\right) $$

### Animals and experimental design

The employed male albino rats (100 g average weight) were purchased from the animal house of Faculty of Agriculture, Minia University, Minia, Egypt. The animals were housed under standardized environmental conditions, with free access to standard diet and water and allowed to acclimate to the environment for one week prior to inclusion in the experiment. Animal experiments were conducted following the guidelines for the care and use of laboratory animals of the National Institutes of Health (NIH publication No. 85–23, revised 1985). The study protocol (code number of project 2015:03) was approved by members of “The Research Ethics Committee” and by the Pharmacology and Toxicology Department, Faculty of Pharmacy, Minia University, Egypt.

### Toxicity study of Salix subserrata

The study was performed over a period of 28 days using 80 rats, randomized into 8 groups of 10 animals each (5 males and 5 females). Group I received a daily oral dose of 5 % carboxymethyl cellulose (CMC). The animals in all other seven groups (group II through VIII) received a daily oral dose of SFEE diluted in 5 % CMC at different concentrations (50, 100, 150, 250, 500, 750 and 1000 mg/kg) to test the safety of the ethanolic extract of *Salix subserrata*.

### Hepatoprotective effect of Salix subserrata

The study was performed over a period of 9 weeks using 40 rats, randomized into 4 groups (group I through IV) of 10 animals each.

- **Group I:** the normal healthy control group: ten rats received olive oil intraperitoneally (i.p.) twice weekly for the whole period of the experiment (9 weeks) along with a daily oral dose of 5 % CMC.
- **Group II:** rats intoxicated with CCl_4_ i.p. (0.8 ml/kg body weight CCl_4_/olive oil, 1:1 v/v, twice weekly) over the whole period of the experiment to induce chronic liver injury [18].
- **Group III:** (SFEE-treated): rats received CCl_4_ i.p. as explained in group II along with a daily oral dose of SFEE (150 mg/kg, diluted in 5 % CMC).
- **Group IV:** (Silymarin-treated): rats received CCl_4_ i.p. as explained in group II along with a daily oral dose of silymarin (100 mg/kg, diluted in 5 % CMC) [19].

In groups III and IV, the treatment with SFEE or silymarin was initiated 24 h after the first dose of CCl_4_.

### Sample collection

Blood samples were collected for biochemical analysis, and liver tissues were excised rapidly and prepared for histological investigation. Blood samples were left for 15 to 30 min for *in vitro* coagulation and then centrifuged at 3,000x g for 15 min in order to collect serum.

### Liver specimen preparation

Each liver specimen was dissected into 2 parts. One part was fixed and embedded in paraffin for histopathological examination. The second part was homogenized for total protein extraction in 20 mM Tris, 100 mM NaCl, 1 mM EDTA and 0.5 % Triton X-100 buffer. Protein content of the different liver homogenates was determined using Biuret reagent and bovine serum albumin as standard. After adding the protease inhibitors mix, homogenates were divided in aliquots and stored at −70 °C until use.

### Western blot analysis

Western blot analysis was performed as described elsewhere [20]. Briefly, 50 μg of total protein from each liver homogenate were denatured by boiling for 5 min in 2 % SDS and 5 % B-mercaptoethanol and loaded into separate lanes of the 12 % gel SDS–PAGE was performed at average 100 volts for 2 h then electro-transferred to a *Hybond*™ nylon membrane (GE Healthcare) using T-77 ECL semi-dry transfer unit (Amersham Biosciences), for 2 h. The membrane was blocked in TBS buffer containing 0.05 % Tween and 5 % non-fat milk for one hour followed by the incubation with rabbit polyclonal anti rat TNF-α (ab 9755) or rabbit polyclonal anti rat NF**k**B (ab 16502) as primary antibodies. Polyclonal goat anti-rabbit or anti-mouse immunoglobulin conjugated to alkaline phosphatase (Sigma–Aldrich, Schelldorf, Germany) diluted 1:5000 in the 10x diluted blocking buffer served as secondary antibody. Protein bands were detected by incubating the membranes with alkaline phosphatase buffer (100 mM tris pH 9.5; 100 mM NaCl; 5 mM MgCl_2_) containing substrate (6.6 μl NBT/ml and 3.3 μl BCIP/ml from stock of 50 mg/mL nitroblue tetrazolium (NTB) and 50 mg/ml 5-bromo-4-chloro-3-indolyl phosphate (BCIP) in 70 % formamide). Color reactions were stopped by rinsing with stop buffer (10 mM Tris-Cl, pH 6.0, 5 mM EDTA) [21].

### Assessment of serum liver function tests, lipid peroxides and hepatic Glutathione content

The biochemical markers of hepatic damage including serum ALT, AST [22], ALP [23], albumin [24], total bilirubin [25], triglycerides (TG) [26], urea [27], creatinine [28], total cholesterol [29], lipid peroxides [30, 31], and GSH content [32] were estimated according to previously reported methods using available commercial kits following manufacturer’s instructions.

### Enzyme-linked immunosorbent assay (ELISA)

LDH was measured using commercially available ELISA kits according to the manufacturer’s instructions [33].

#### Histopathological investigation

Formalin-fixed liver specimens were prepared from four, randomly chosen rats per group. Specimens were dehydrated in a series of increasing ethanol concentrations then embedded in paraffin. Tissue sections (5 μm) were stained with haematoxylin and eosin (HE). At least three slides were prepared from each specimen and blindly examined. Histopathological scoring was achieved via an expert pathologist using METAVIR scoring, using Optica B-82 microscope for detection of pathological changes.

### Statistical analysis

Data were expressed as the mean ± standard error of the mean (SEM) and were analyzed for statistically significant differences using one-way analysis of variance (ANOVA) followed by the Tukey-Kramer post analysis test to compare all groups. Kruskal-Wallis non-parametric test followed by Dunn’s multiple comparison post hoc test, was used for analysis of histological scoring. P values less than 0.05 were considered as significant. GraphPad Prism® was used for statistical calculations (Version 5.00 for Windows, GraphPad Software, San Diego California USA, www.graphpad.com).

## Results

### In vitro radical scavenging activity of SFEE

**Fig. 1:**
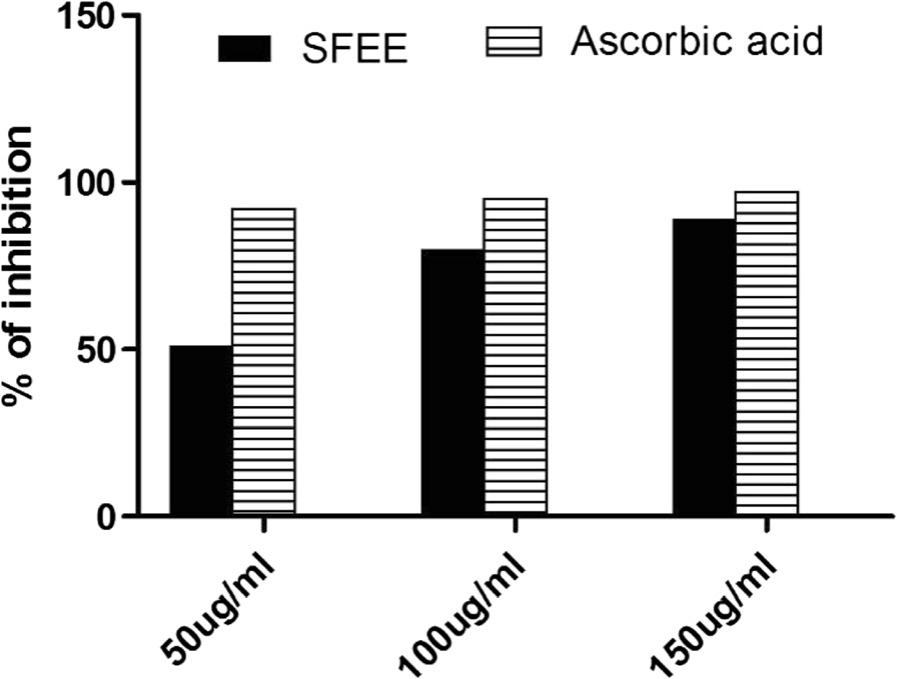
DPPH scavenging activity of SFEE compared to ascorbic acid as a known DPPH radical scavenger (*n* = 3)

DPPH assay is one of the commonly used method to evaluate the free radical scavenging activity of antioxidants where the reduction in DPPH absorbance correlates directly with the antioxidant activity. In this study, the antioxidant activity of different concentrations of SFEE was estimated using DPPH in comparison to ascorbic acid as a standard antioxidant. SFEE at a concentration of 150 ug/ml showed an 80–90 % inhibition, which was very close to the effect of ascorbic acid at the same concentration (Fig. 1)

### Toxicity studies for the SFEE

Seven groups of rats administered 50, 100, 150, 250, 500, 750 and 1000 mg/kg of SFEE orally to estimate the safe dose of the SFEE. No signs of toxicity or behavioral changes were observed in test group in comparison to the healthy control group [34]. Moreover, all of the previously mentioned biochemical parameters including liver enzymes and other liver biomarkers together with creatinine and urea were measured (Table 1). The values obtained for these parameters revealed that up to 1000 mg of SFEE can be regarded as a safe dose compared to the healthy control group based on the unchanged values of the liver as well as kidney function markers. This proofs the wide margin of safety of *Salix subserrata*.

**Table 1:** Assessment of orally-administered SFEE on serum biochemical parameters in rats treated for 28 days

	Group I	Group II	Group III	Group IV	Group V	Group VI	Group VII	Group VIII
	Control	50 mg/kg	100 mg/kg	150 /kg	250 mg/kg	500 mg/kg	750 mg/kg	1000 mg /kg
Albumin (mg/dl)	4.69 ± 0.12	4.65 ± 0.1	4.72 ± 0.27	4.63 ± 0.16	5.1 ± 1.2	4.53 ± 0.26	4.87 ± 0.19	4.92 ± 0.19
Cholesterol (mg/dl)	72 ± 3.07	73 ± 2.09	71 ± 3.6	71 ± 3.1	72 ± 5.7	73 ± 1.2	70 ± 1.7	74 ± 6.1
Bilirubin (mg/dl)	0.11 ± 0.03	0.12 ± 0.01	0.11 ± 0.02	0.11 ± 0.02	0.11 ± 0.02	0.13 ± 0.06	0.12 ± 0.08	0.13 ± 0.01
Triglycerides (mg/dl)	71 ± 2.5	72 ± 3.1	71 ± 2.3	69 ± 1.6	73 ± 1.5	71 ± 2.1	72 ± 3.2	73 ± 6.1
ALT (U/L)	42 ± 1.3	41 ± 2.3	44 ± 3.5	42 ± 2.6	43 ± 3.6	42 ± 3.1	44 ± 2.5	44 ± 3.1
AST (U/L)	101 ± 2.8	101 ± 3.8	99 ± 5.2	100 ± 3.7	102 ± 5.4	98 ± 3.8	102 ± 5.3	103 ± 7.3
ALP (U/L)	24 ± 2.1	24 ± 1.57	25 ± 1.5	24 ± 2.2	26 ± 1.2	25 ± 1.7	25 ± 1.4	24. ± 2.2
LDH (U/L)	578 ± 37	572 ± 39	587 ± 57	581 ± 48	601 ± 35	590 ± 45	600 ± 53	598 ± 65
GSH (μM/100 mg liver)	5.2 ± 0.87	5.2 ± 0.12	5.4 ± 0.45	5.3 ± 0.58	5.4 ± 0.97	5.1 ± 0.3	5.2 ± 0.4	5.23 ± 0.5
MDA (n mole/g liver)	17 ± 2.9	18 ± 3.2	16 ± 3.8	17.2 ± 4.1	17 ± 3.5	17.8 ± 2.8	18 ± 4.8	19 ± 3.7
Urea (U/L)	5.1 ± 0.68	5.3 ± 0.62	5.2 ± 0.76	5.2 ± 0.13	5.2 ± 0.56	5.3 ± 0.4	5.13 ± 0.34	5.3 ± 0.67
Creatinine (mg/dl)	25 ± 2.3	27 ± 2.1	25 ± 0.83	25 ± 3.1	26 ± 0.76	26.7 ± 0.7	27.1 ± 0.9	28.2 ± 3.7

Values represent Mean ± SEM (*n* = 10)

*ALT* Alanine aminotransferase, *AST* Aspartate aminotransferase, *ALP* Alkaline phosphatase, *GSH* Total thiol, *MDA* Malondialdehyde

All groups compared to group I (healthy control group). No significant difference between group II, through VIII (50, 100, 150, 250, 500, 750 and 1000 mg/kg SFEE respectively) and group I (healthy control), *p* = 0.5 or higher using one-way Anova followed by Tukey-Kramer test

### SFEE treatment restores serum liver enzymes activity

The released biomarkers of liver cell integrity; ALT, AST, ALP and LDH were investigated in the serum of the different groups. Administration of CCl_4_ resulted in an approximately 2.5 folds increase in the mean value of ALT levels in comparison to the healthy control (Table 2). Interestingly, the daily treatment with 150 mg/kg SFEE reversed the elevation in the levels of ALT caused by CCl_4_ resulting in values that were comparable to healthy control and to that produced by silymarin, noting that silymarin is well known for its hepatoprotective action. Notably, this reduction in serum activity of ALT was significant (*P* < 0.001) in comparison to that observed after CCl_4_ treatment (Table 2). A similar tendency was also observed in case of AST, ALP and LDH enzyme levels as shown in Table 2. Lower doses of SFEE (50 and 100 mg/kg) were unable to restore normal values of serum lever enzymes (data not shown).

**Table 2:** Effect of SFEE on the levels of liver biomarkers compared to silymarin after CCl_4_ intoxication

	Group I	Group II	Group III	Group IV
	Control	CCl4	CCl4 + SFEE (150 mg/kg)	CCl4 + Silymarin (100 mg/kg)
Albumin (mg/dl)	4.7 ± 0.034	3.2 ± 0.45^+++^	4.7 ± 0.087**	4.5 ± 0.17**
Cholesterol (mg/dl)	72 ± 3.1	130 ± 12^+++^	72 ± 2.5***	68 ± 3.5***
Bilirubin (mg/dl)	0.11 ± 0.045	0.39 ± 0.03^+++^	0.11 ± 0.031***	0.087 ± 0.033**
Triglyceride (mg/dl)	71 ± 4.1	130 ± 6.9^+++^	80 ± 6.8***	75 ± 6.7***
ALT (U/L)	40 ± 3.1	100 ± 6.9	41 ± 2.4***	42 ± 2.2***
AST (U/L)	100 ± 5.9	240 ± 23	110 ± 5.1***	110 ± 4.0***
ALP (U/L)	24 ± 1.9	160 ± 10.6^+++^	28.5 ± 2.1***	26 ± 1.4***
LDH (U/L)	570 ± 42	1200 ± 56^+++^	580 ± 52***	600 ± 59***

Values represent Mean ± SEM (*n* = 10)

*ALT* Alanine aminotransferase, *AST* Aspartate aminotransferase, *ALP* Alkaline phosphatase, *GSH* Total thiol, *LDH* Lactate dehydrogenase

***p < 0.001 (compared to CCl4 (group II)) and ^+++^ *p* < 0.001 (compared to control group I). No significant difference (*p* = 0.3 or higher) between group III (150 mg/kg SFEE) and group IV (silymarin), using one-way ANOVA test followed by Tukey-Kramer test

**p < 0.01

### SFEE treatment restores cholesterol, TG, albumin and bilirubin levels

Table 2 shows the change in the serum levels of cholesterol, TG, bilirubin and albumin following the different treatments. The administration of CCl_4_ resulted in a significant elevation in the serum levels of cholesterol, TG and bilirubin (*P* < 0.001) together with a significant reduction in albumin level (*P* < 0.001). Interestingly, SFEE treatment was significantly capable of preventing the elevation in serum levels of total cholesterol, TG and bilirubin induced by CCl_4_ (*P* < 0.001).

### SFEE treatment restores GSH level

**Fig. 2:**
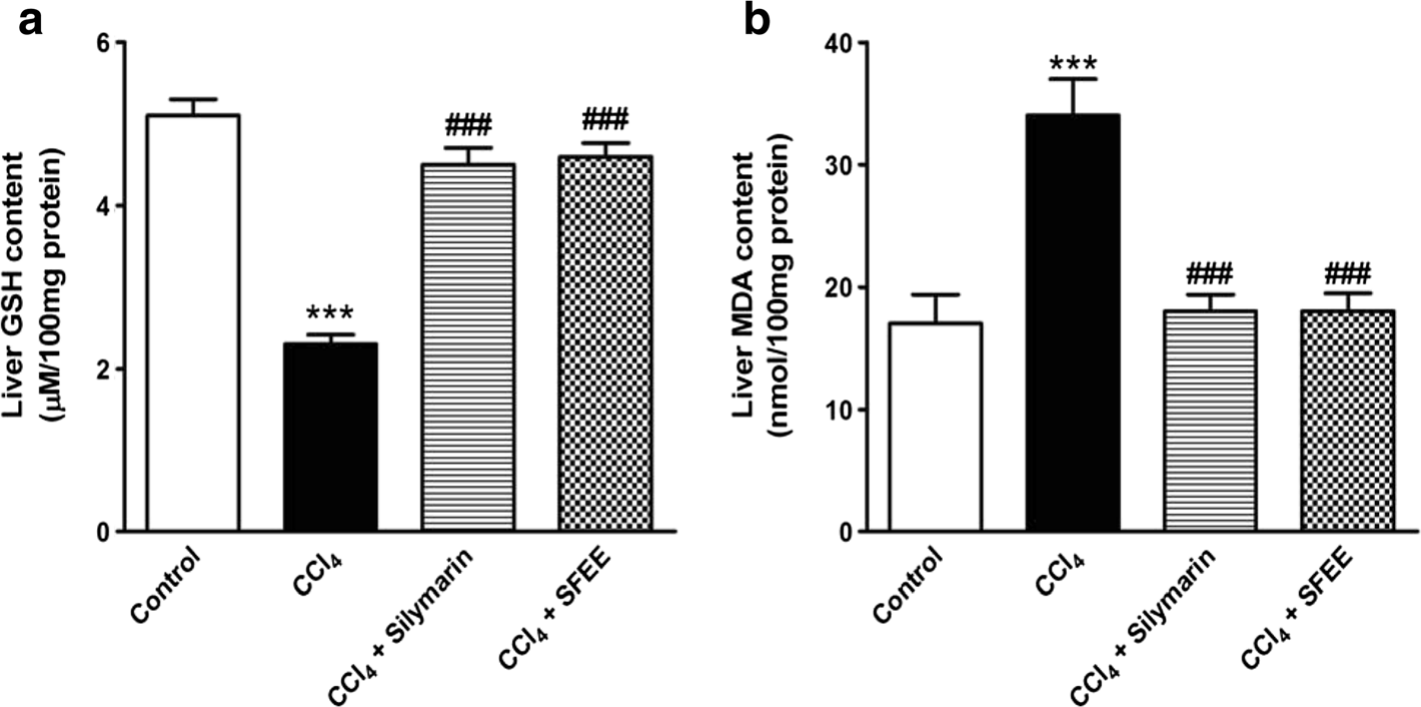
Effect of SFEE treatment on the total hepatic thiol content and lipid peroxidation. **a**. SFEE treatment resulted in a significant improvement in the hepatic GSH content compared to CCl_4_ treated animals (*p* < 0.005). No significant difference could be detected among SFEE-treated group, silymarin-treated group and control (*n* = 10, *p* > 0.3). **b**. SFEE treatment resulted in normalization of lipid peroxidation (measured as MDA) despite CCl_4_ co-administration (*n* = 10, *p* < 0.05). Data are expressed as mean ± SEM, significance was calculated using one-way ANOVA followed by Tukey-Kramer multiple comparisons post hoc test

The hepatic tissue content of GSH was measured in the healthy control, CCl_4_, silymarin/CCl_4_ and SFEE/CCl_4_-treated groups as shown in Fig. 2a. Observed values revealed a significant reduction in the hepatic GSH level in the group of rats treated with CCl_4_ (*p* < 0.005) when compared to healthy control rats (5.05 μM GSH/100 mg total protein for the healthy control compared to 2.29 μM GSH/100 mg protein for the CCl_4_-treated group). Notably, SFEE treatment effectively prevented the CCl_4_-induced depletion of GSH content of liver caused by CCl_4_ administration. The effect obtained by SFEE treatment was comparable to that observed in silymarin/CCl_4_-treated groups (*P* > 0.3).

### SFEE treatment prevents lipid peroxidation

Malondialdehyde (MDA) is one of the final products of lipid peroxidation and is considered as one of its markers. In the current study, we found that CCl_4_ treatment of animals resulted in a significant, 3.12 folds, increase in the tissue level of MDA (*P* < 0.001) compared to healthy control animals as expected (Fig. 2b). To our interest, SFEE treatment resulted in a significant prevention of the CCl_4_-induced overexpression of MDA (*P* < 0.001) compared to the CCl_4_-treated group. The values observed following SFEE/CCl_4_ treatment were comparable to those observed in healthy control as well as silymarin/CCl_4_-treated groups.

### SFEE treatment decreases the expression levels of TNF-α and NF-**k**B proteins in CCl_4_ intoxicated rats

**Fig. 3:**
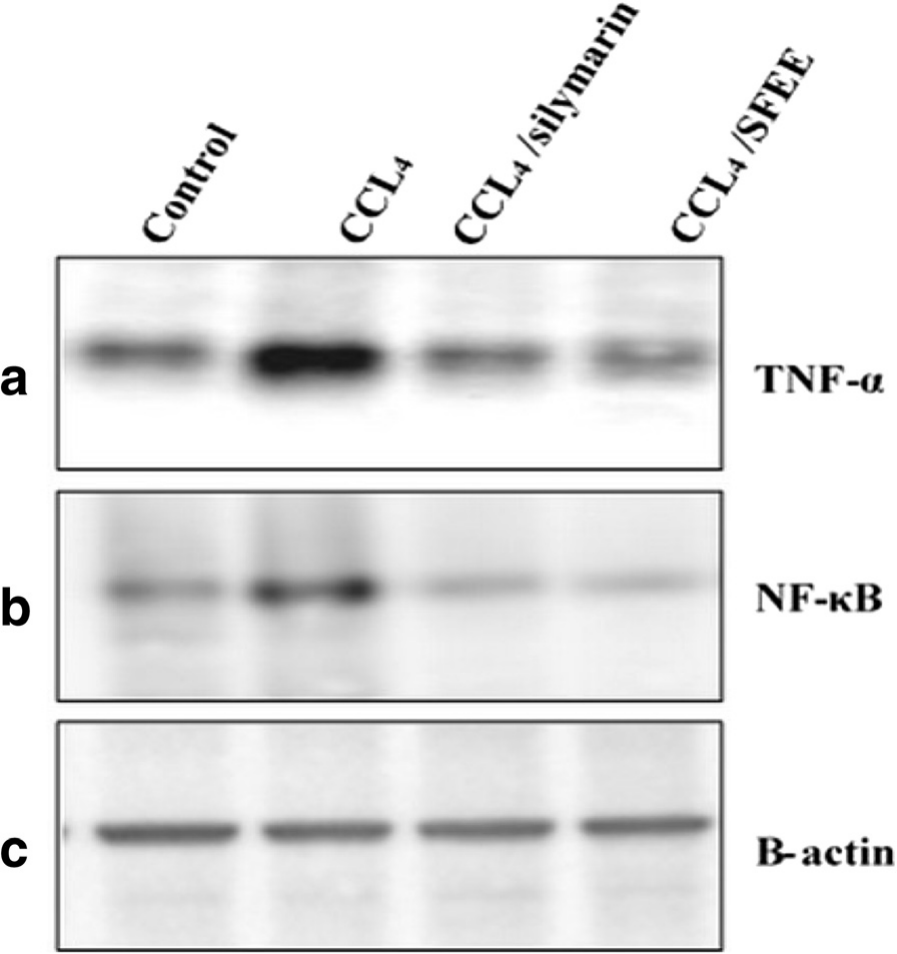
Effect of SFEE on TNF-α and NF-kB protein expression level. Panel **a**: TNF-α; panel **b**: NFkB and panel **c**: Anti-β actin antibodies (internal loading control). Lane 1: Control untreated rats receiving only olive oil, Lane 2: rats receiving 0.8 ml/kg CCl_4_ twice weekly, Lane 3: rats receiving 100 mg/kg silymarin/CCl_4_ and Lane 4: rats receiving 150 mg/kg of SFEE/CCl_4_. CCL_4_ treatment resulted in an elevated expression of both TNF-α and NF-kB whereas their expression was normalized after SFEE as well as silymarin treatment

The levels of the TNF-α and NF**k**B proteins were assessed using the western blot technique in the different experimental groups. As shown in Fig. 3, CCl_4_ treatment resulted in over expression of TNF-α and NF**k**B, when compared to healthy control and silymarin/CCl_4_ treated groups. Interestingly, treatment with SFEE resulted in a reduced expression of TNF-α and NF-**k**B, when compared to that of CCl_4_ treatment (Fig. 3).

### Histopathological findings of the SFEE/CCl_4_-treated group

**Fig. 4:**
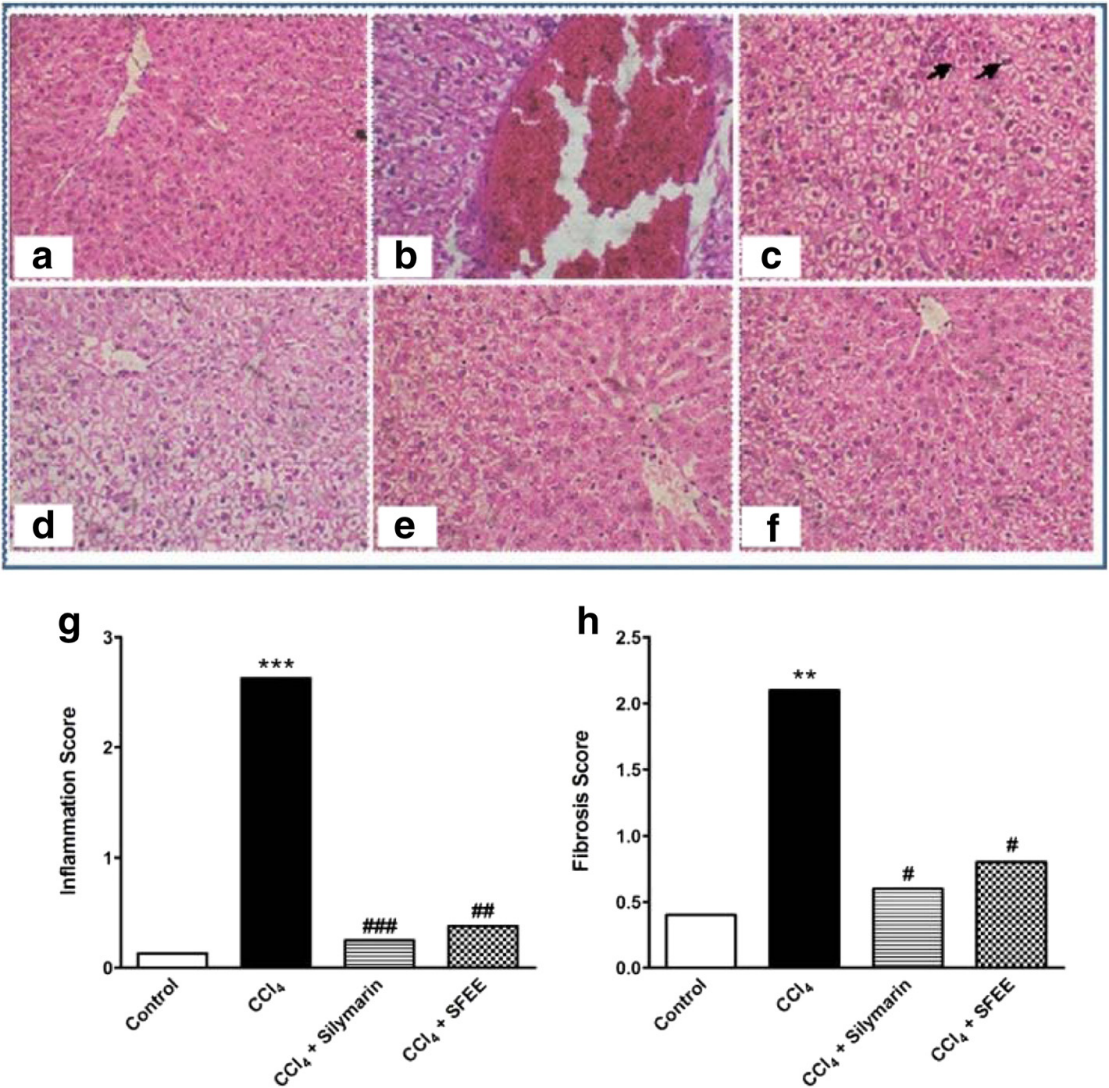
Histological examination of liver sections from different groups. Liver sections from healthy control show normal hepatocytes architecture (**a**), whereas CCl_4_ treatment resulted in damaged cells, shrunken nuclei, mitotic activity (arrow heads) and centrilobular congestion (**b** & **c**). SFEE treatment resulted in restoration of the normal architecture and absence of congestion (**e** & **f**) in a similar way to that observed in silymarin treatment (**d**). Bars represent mean ± SEM of histopathological scoring (**g**) inflammation score and (**h**) fibrosis score. #, *: significantly different compared to CCl_4_-treated group or control group respectively, *p* < 0.05. Significance was calculated using Kruskal-Wallis test followed by Dunn’s multiple comparison post hoc test

To assess the effect of the different treatment protocols on liver architecture, paraffin section prepared from the hepatic tissues of the different groups were stained with hematoxylin/eosin and examined. From the histological point of view, liver from rats in the healthy control group showed a normal liver lobular architecture and hepatocyte structure (Fig. 4a). In contrast, CCl_4_ administration resulted in histopathological lesions and extensive hepatocellular damage, as represented by the presence of portal inflammation, fatty change and venous congestion (Fig. 4b, c). Treating the tested animals with SFEE was capable of ameliorating these histopathological changes (Fig. 4e, f), producing similar effects to that achieved by the treatment with Silymarin (Fig. 4d). SFEE as well as silymarin were able to significantly decrease the signs of CCl4 -induced toxicity (*P* < 0.05) (Fig. 4g, h).

## Discussion

Metabolism and excretion of xenobiotics usually result in the generation of free radicals, which eventually causes damage to the hepatic parenchyma (hepatocellular damage). Such damage is shown to be caused by a number of drugs and viral infections [35].

In light of the limited pharmacological options available for the treatment of liver diseases, identification of effective hepatoprotective agents derived from natural sources is an urgent necessity. Therefore, it is important to evaluate plant extracts that can help in restoring liver functions.

Since ancient times, natural products such as herbs have been used as a remedy for various diseases. Indeed, plant extracts usually possess variable amounts of phenolic and polyphenolic compounds, which are responsible for the antioxidant effects of these medicinal plants [15]. Therefore, we sought to investigate the effect of SFEE as a possible hepatoprotective agent.

The CCl_4_-intoxicated rat animal model has been widely used for decades to investigate the mechanisms of acute and chronic liver injuries depending on the dose and frequency of injection. Based on CCl_4_ hepatocellular damaging effect, this model has also been widely used as the most reliable, best characterized system for screening hepatoprotective drugs. This experimental model involves the formation of free radicals which are metabolized in the liver producing highly reactive and lethal trichloromethyl free radicals (CCl_3_). These free radicals are converted to trichloromethyl peroxy radical (CCl_3_OO^.^) via the cytochrome P450 oxygenase enzyme resulting in a condition of oxidative stress. This initiates autoxidation of lipids via binding to polyunsaturated cytoplasmic membrane fatty acids, leading to cellular membrane damage and eventually liver diseases [4, 5, 36]. The body has several mechanisms to counteract oxidative stress induced by CCl_4_ with the aid of naturally existing “endogenous” antioxidants, or “exogenous” antioxidants that can be supplemented in the diet. Antioxidants neutralize excess free radicals and hence protect cells against their toxic effects. Among these antioxidants are polyphenolic-containing drugs such as SFEE.

The toxicity was evaluated for seven different doses of SFEE, and was assessed based on the changes in liver as well as kidney biomarkers. Interestingly, the investigated parameters revealed the safety of the whole set of doses of SFEE used in the experiment compared to the healthy control group.

Both TNF-α and NF-**k**B are used as inflammatory biomarkers. Whereas TNF-α is a pro-inflammatory cytokine that is involved in central inflammation processes, NF-**k**B is an inducible, transcription factor that regulates the expression of genes involved in the inflammation process.

TNF-α has a role in regulating a wide range of physiological events, including apoptosis and inflammatory processes [37], as well as its role in other diseases such as diabetes [38, 39]. It has been previously reported that inflammatory cytokine activity is increased in many forms of experimental and clinical forms of liver injury [40–44].

NF-**k**B is a heterodimeric protein that is retained in the cytoplasm in an inactive form by binding to I**k**B (inhibitor of NF-**k**B). Upon induction, NF-**k**B is unbound from I**k**B and translocated to the nucleus, where it binds to DNA and activates transcription [45, 46].

In the current study, CCl_4_-treated rats showed severe inflammation and hence an overexpression of TNF-α. As observed in our results, SFEE treatment effectively prevented CCl_4_-induced liver injury which can then be explained by ameliorating the inflammatory process via normalizing the levels of the inflammatory mediators TNF-α and NF-**k**B proteins. This protective effect was comparable to that of the standard hepatoprotective agent silymarin.

In the current study, silymarin was used as a control for its well-known hepatoprotective action. It is a flavonoid complex, that is obtained from *Silybum marianum*, known as milk thistle, belonging to family Asteraceae (=Compositae) [47, 48]. The hepatoprotective effects of this natural product have been previously attributed to its antioxidant properties [47, 48].

GSH, the major non-protein thiol in body tissues, is considered the main detoxifying and antioxidant molecule produced by cells. It becomes conjugated to foreign compounds to eliminate their toxic effects. Therefore, measuring its level in the liver provides an indication about the extent of cell damage caused by a certain compound. Indeed, GSH has a central role in protecting cells against the damage resulting from CCl_4_ intoxication by covalently binding to the free radicals produced as a result of CCl_4_ metabolism. In absence of proper antioxidants, these free radicals would initiate a chain reaction that results in lipid peroxidation of cellular membranes and eventually cell membrane disruption, changing cellular membrane fluidity and permeability [49–51]. These protective effects of GSH cause it to be a crucial indicator of chronic injuries in liver tissues. In the current study, we showed that CCl_4_ injection produced a significant depletion in hepatic GSH content, which goes in accordance with previous reports that showed the same GSH-depleting effects for CCl_4_ [52]. Notably, treating rats with SFEE significantly prevented the depletion of hepatic GSH content that would result from CCl_4_ intoxication, which suggests antioxidant properties for this extract.

As mentioned, CCl_4_ can cause liver injury when its free radicals combine with polyunsaturated fatty acids (PUFAs) in hepatic cellular membranes, resulting in their peroxidation. This process results in the elevation of thiobarbituric acid reactive substances (TBARS) which is a major reactive aldehyde resulting from the peroxidation of PUFAs [53–55]. In the current study, TBARS level was notably increased in CCl_4_-treated rat liver compared to that of the healthy control group, indicating CCl_4_-induced oxidative stress. Whereas SFEE treatment decreased TBARS production in the CCl_4_-treated rat liver homogenates. In other words, SFEE partly attenuated oxidative stress by decreasing lipid peroxidation in CCl_4_-treated rats. This led us to conclude that this effect can be attributed to the powerful antioxidant and free radical scavenging activities of the extract.

Hemoglobin is normally degraded into bilirubin and is normally excreted into bile. Following severe liver injury, less bilirubin will be excreted resulting in hyperbilirubinemia, which reflects liver damage (necrosis) [56]. The increase in total serum bilirubin concentration following CCl_4_ administration can be explained by the failure of the damaged hepatic parenchyma to bind, conjugate and excrete the produced bilirubin. Notably, SFEE treatment prevented the elevation of serum bilirubin level compared to the CCl_4_-treated group. These results indicate an improvement in the liver secretory function following administration of the extract.

Serum liver enzyme levels have been widely recognized as crucial biomarkers for the severity of hepatocellular damage. Estimating the serum level of such enzymes provides a reliable image for structural integrity of liver cells. Their serum level reflects the extent of liver damage, as the loss of liver cell structural integrity leads to an increase in the serum level of such enzymes that are typically located in the cytoplasm.

As discussed above, the presence of excessive amounts of free radicals as a result of CCl_4_ administration damages liver cell membranes. As a result, it is expected that cytoplasmic liver enzymes like ALT, AST and ALP will leak into the blood stream in amounts that are relative to the extent of liver damage [49–51]. The normalization of the serum activity of such enzymes following the administration of SFEE can be linked to its effect on healing and regeneration of the hepatocytes.

Our data presented an elevation of serum cholesterol and triglycerides as a result of CCl_4_-induced liver damage, which goes in accordance with previous reports [57]. Interestingly, treating animals with SFEE resulted in a significant improvement of serum lipid profile.

LDH enzyme is an oxidoreductase enzyme that catalyzes the interconversion of pyruvate and lactate in the liver in addition to a number of other body tissues. As in the case of other liver enzymes, serum LDH is increased following liver damage and is hence used as a biomarker for evaluating the degree of liver injury. In line with a previous study from our lab [58], SFEE decreased the LDH level in liver extracts in a similar way to that observed for the other liver enzymes.

The normalization of elevated levels of serum enzymes, as observed after SFEE administration, is an indication of the stabilization of plasma membranes and the reversal of hepatic tissue damage caused by CCl_4_. This SFEE-stabilizing effect on plasma membranes can explain the regain of normal serum activities of liver enzymes in CCl_4_-induced liver damage after the treatment. We attribute the reason behind this to the antioxidant activity of SFEE, which blocks, at least in part, the effects of released free radical metabolites of CCl_4_ that leads to lipid peroxidation and hence membrane destabilization and eventually liver cell injury.

## Conclusion

SFEE showed potential hepatoprotective effects against chronic liver injury, which is likely due to its antioxidant and anti-inflammatory properties. These effects, at least in part, prevent CCl_4_ free radical derivatives formation and hence inhibit cellular damage. Our data are in line with previous reports emphasizing the high antioxidant activity of SFEE due to its high content of phenolic compounds that possess high radical quenching abilities. Accordingly, our findings may play a role towards the discovery of a new naturopathic remedy.

## Abbreviations

ALP, alkaline phosphatase; ALT, alanine aminotransferase; AST, aspartate aminotransferase; BCIP, 5-bromo-4-chloro-3-indolyl phosphate (BCIP); CCl_4_, carbon tetrachloride; CMC, carboxymethyl cellulose; DPPH, 2.2-Diphenyl-1-picryl hydrazyl; EDTA, Ethylenediaminetetraacetic acid; GSH, total glutathione; H&E, hematoxylin-eosin; LDH, lactate dehydrogenase; MDA, malondialdehyde; NF-kB, nuclear factor kappa-B; NBT, nitroblue tetrazolium; PUFAs, polyunsaturated fatty acids; ROS, reactive oxygen species; SE, standard error; SFEE, *Salix subserrata* Willd flower ethanolic extract; TBARS, thiobarbituric acid reactive species; TNF-α, tumor necrosis factor-alpha
